# Organizational Climate, Role Conflict, and Job Esteem Among Registered Nurses in Physician-Delegated Roles in South Korea: A Cross-Sectional Study

**DOI:** 10.3390/healthcare14101368

**Published:** 2026-05-16

**Authors:** Youngeun Lee, Gaeun Kim

**Affiliations:** 1 Cardiac Intensive Care Unit, Keimyung University Dongsan Hospital, Daegu 42601, Republic of Korea; 2 College of Nursing, Keimyung University, 1095 Dalgubeol-Daero, Dalseo-Gu, Daegu 42601, Republic of Korea

**Keywords:** physician assistant nurse, organizational climate, role conflict, job esteem, nursing workforce, healthcare policy

## Abstract

Background/Objectives: In South Korea, physician assistant (PA) nurses—registered nurses performing physician-delegated advanced clinical tasks without a nationally standardized licensure system—are increasingly relied upon to address healthcare delivery gaps. This study examined the associations of organizational climate and role conflict with job esteem among PA nurses. Methods: A cross-sectional descriptive design was used. Data were collected from 145 PA nurses at four university hospitals (each ≥ 500 beds) in Daegu, South Korea, between March and April 2025, using validated instruments for organizational climate, role conflict, and job esteem. Multiple linear regression analysis was performed to identify factors associated with job esteem. Results: Organizational climate was positively correlated with job esteem, while role conflict showed a negative correlation. In the multiple linear regression model, organizational climate and pre-role training experience emerged as significant factors associated with job esteem, jointly explaining 23% of the variance, whereas role conflict did not show an independent association when organizational climate was included in the model. Conclusions: These findings suggest that supportive organizational climates and structured preparation may be important for sustaining job esteem among nurses working in expanded physician-delegated roles. In the broader context of physician shortages, which can compromise care quality and intensify nurses’ workload, strengthening organizational supports for PA nurses is also relevant to maintaining the quality and continuity of healthcare services. These findings may also be informative for other healthcare systems in which nursing role expansion is occurring faster than the development of supporting institutional structures.

## 1. Introduction

Healthcare systems worldwide are confronting workforce sustainability challenges driven by physician shortages, aging populations, and the need for cost-effective care delivery models [1,2]. A common policy response has been to expand the clinical roles of nurses—through various advanced practice designations—shifting tasks traditionally performed by physicians to nursing professionals [3,4]. However, the speed and scope of such role transitions vary considerably across countries, and the institutional supports—legal protections, standardized education, and organizational recognition—often lag behind operational demands [5]. This gap between expanded responsibilities and inadequate institutional backing may undermine the professional well-being of the very workforce these systems depend upon. In contexts where role expansion occurs faster than legal and educational reform, organizational conditions may become especially important determinants of professional well-being.

South Korea presents a particularly instructive case. Following the 2024 healthcare reform conflict that led to mass resignations among resident physicians, the government rapidly expanded nursing tasks through a pilot project defining the scope of physician-delegated duties [6]. Yet the approximately 10,000 physician assistant (PA) nurses working in Korean hospitals continue to operate without a clear legal mandate, standardized scope of practice, or nationally accredited training programs [7,8]. It is important to note that the term “PA nurse” as used in this study refers specifically to registered nurses in South Korea who perform physician-delegated advanced clinical tasks—including wound management, procedural assistance, order entry, and patient assessment—within hospital settings, without a nationally standardized PA licensure system. This role differs substantially from the US Physician Associate/Physician Assistant, which is a distinct licensed healthcare profession with its own graduate-level education and national certification [4]. The Korean PA nurse role also differs from formally credentialed Advanced Practice Nurse (APN) or Nurse Practitioner (NP) designations found in other countries [3,9]. This institutional ambiguity is not unique to Korea; similar challenges have been documented in other rapidly transitioning healthcare systems where task-shifting policies outpace regulatory frameworks [1,5].

Job esteem—defined as the beliefs, values, and evaluative perceptions individuals hold regarding their professional worth [10]—is increasingly recognized as a relevant factor in nursing workforce sustainability. Unlike job satisfaction, which reflects affective responses to the job itself, job esteem captures the deeper normative judgment about the social value and professional identity of one’s work. Higher job esteem has been associated with increased retention intention [11], reduced turnover intention [12], greater organizational commitment [13], and improved nursing performance [14]. Given that PA nurses constitute a growing and essential segment of the hospital workforce, understanding the factors associated with their job esteem is important not only for individual well-being but also for organizational performance and patient safety.

Two organizational factors are particularly relevant for PA nurses: organizational climate and role conflict. Organizational climate encompasses nurses’ shared perceptions of the policies, practices, and procedures within their work setting, including autonomy, interprofessional collaboration, administrative support, and professional visibility [15]. In the present study, we use the term “organizational climate” (rather than the broader term “work environment” that is sometimes used interchangeably in the nursing literature) because the variable was operationalized using the Nurse Practitioner Primary Care Organizational Climate Questionnaire (NP-PCOCQ), which directly assesses dimensions of organizational climate as defined by Poghosyan et al. [15] and adapted for Korean PA nurses by Yu et al. [16]. Favorable practice environments have been linked to higher job satisfaction, stronger professional identity, and better patient outcomes across diverse healthcare settings [17,18]. For PA nurses, whose role boundaries are often undefined, the organizational climate may serve as a critical resource that shapes their sense of professional worth [16].

Role conflict—arising when expected roles within an organization are contradictory, unclear, or incompatible with available resources [19]—is a well-documented challenge for PA nurses. In the absence of standardized scope-of-practice definitions, PA nurses frequently navigate ambiguous task boundaries, conflicting expectations from physicians and nursing managers, and responsibilities that exceed their formal authority [7,20,21]. Such conflict has been associated with job burnout, increased turnover intention, and diminished professional identity [19,22]. However, how role conflict relates to job esteem—and whether its association is independent of or overlaps with organizational climate—remains underexplored.

A previous study by Cho [23] examined the associations of emotional intelligence and organizational climate with job esteem among PA nurses, identifying both as significant factors. However, that study focused primarily on individual psychological factors without incorporating organizational-structural variables such as role conflict. In addition, prior Korean studies have examined related but distinct outcomes such as turnover intention [21], burnout [19], and job satisfaction [24] among PA nurses, identifying role conflict and organizational conditions as important contributing factors. International studies on advanced practice nurses have similarly reported that supportive practice environments are associated with greater job satisfaction and intent to stay [23], while unresolved role ambiguity has been linked to diminished professional well-being [22]. Despite this growing evidence base, few studies have simultaneously examined organizational climate and role conflict in relation to job esteem specifically among Korean PA nurses, leaving an important gap in our understanding of how these organizational factors jointly shape professional self-regard. Given that PA nurses face simultaneous challenges from both environmental and role-structural domains [25], a more comprehensive model incorporating organizational factors is needed. Examining both organizational climate and role conflict may therefore help clarify whether job esteem is shaped more by supportive organizational conditions or by the strain of unresolved role ambiguity.

Therefore, this study aimed to examine the associations of organizational climate and role conflict with job esteem among PA nurses in South Korea. Specifically, the study sought to: (1) describe the levels of organizational climate, role conflict, and job esteem among PA nurses; (2) identify differences in job esteem according to demographic and job-related characteristics; (3) examine correlations among organizational climate, role conflict, and job esteem; and (4) identify factors significantly associated with job esteem. By doing so, this study seeks to generate evidence relevant both to internal organizational expectations within Korean hospitals regarding the management of PA nurses and to the broader international discourse on workforce governance during role boundary transitions.

## 2. Materials and Methods

### 2.1. Study Design

A cross-sectional descriptive study was conducted to examine the associations of organizational climate and role conflict with job esteem among physician assistant (PA) nurses.

### 2.2. Participants and Setting

A convenience sample was recruited from four tertiary university hospitals (each ≥ 500 beds) in Daegu Metropolitan City, South Korea. These were teaching hospitals with diverse medical departments providing advanced medical care and residency training. The eligible target population comprised registered nurses currently performing physician-delegated advanced clinical tasks (e.g., wound management, procedural assistance, order entry, patient assessment) at one of the four participating hospitals during the recruitment period. The inclusion criteria were: (1) currently working in a PA role at one of the four participating hospitals; (2) having at least one year of total clinical nursing experience, as prior research indicates that at least one year is generally required for organizational adaptation and independent task performance [26]; and (3) being able to read and understand Korean and willing to provide written informed consent. Nurses with less than one year of total clinical experience were excluded. A formal enumeration of the total number of PA nurses across the four institutions was not undertaken in this study; instead, the recruitment strategy was designed to reach all eligible nurses through both department bulletin boards and internal hospital messaging systems during the recruitment period. All PA nurses meeting the inclusion criteria at the participating hospitals were invited to participate on a voluntary basis.

The minimum sample size was calculated using GPower 3.1.9.7 for multiple linear regression (significance level α = 0.05, power 1 − β = 0.80, medium effect size f^2^ = 0.15, 14 predictors), yielding a requirement of 139 participants. The GPower 3.1.9.7 (Heinrich-Heine-Universität Düsseldorf, Düsseldorf, Germany) calculation was based on the maximum number of candidate predictors considered at the study design stage, which included all demographic and job-related variables that could potentially enter the model; in the final regression, a smaller set of variables was retained based on the results of bivariate analyses and theoretical considerations. Accounting for a 10% anticipated dropout, 154 questionnaires were distributed, and 145 valid responses (response rate: 94.2%) were retained after excluding 9 incomplete surveys.

### 2.3. Instruments

#### 2.3.1. Organizational Climate

Organizational climate was measured using the Nurse Practitioner Primary Care Organizational Climate Questionnaire (NP-PCOCQ), originally developed by Poghosyan et al. [15] and adapted for Korean PA nurses by Yu et al. [16]. The instrument contains 29 items across four subscales: professional visibility (4 items), relationships with administrators (9 items), relationships with medical staff (7 items), and independent practice and support (9 items). Each item was rated on a 4-point Likert scale (1 = strongly disagree, 4 = strongly agree), with higher scores indicating a more favorable organizational climate. Cronbach’s α was 0.90 in the original study [15], 0.93 in Yu et al. [16], and 0.92 in this study.

#### 2.3.2. Role Conflict

Role conflict was measured using the instrument developed by Kim and Park [27] and modified for PA nurses by Lee and Kim [24]. The instrument comprises 34 items across four subscales: role ambiguity (15 items), perceived incompetence (10 items), work overload (4 items), and lack of cooperation (5 items). Items were rated on a 5-point Likert scale (1 = strongly disagree, 5 = strongly agree), with higher scores indicating greater role conflict. Cronbach’s α was 0.95 in the original study [27], 0.97 in Lee and Kim [24], and 0.94 in this study.

#### 2.3.3. Job Esteem

Job esteem was measured using the Job Esteem Scale for Korean Nurses (JES-KN) developed by Choi and Jeong [10]. The instrument contains 28 items across six subscales: professional self-awareness (7 items), professional competence (5 items), care role and expertise (4 items), social trust and respect (4 items), organizational respect and recognition (4 items), and professional authority and future value (4 items). Items were rated on a 5-point Likert scale (1 = strongly disagree, 5 = strongly agree), with higher scores indicating higher job esteem. Cronbach’s α was 0.94 in the original study [10] and 0.90 in this study.

All three instruments were administered in Korean. The NP-PCOCQ had been previously translated and adapted for Korean PA nurses by Yu et al. [16], and the role conflict scale [27] and the JES-KN [10] were originally developed and validated in Korean. Permission to use each instrument was obtained from the developers of the original and the Korean-adapted versions prior to data collection.

### 2.4. Data Collection

Data were collected between 25 March and 9 April 2025. The researcher contacted each hospital’s nursing department to explain the study purpose and procedures and provided official cooperation letters along with the Institutional Review Board (IRB) approval. Recruitment announcements were posted on department bulletin boards and shared through internal messaging systems. Participation was voluntary and anonymous. Participants who provided written informed consent completed self-administered paper questionnaires in private. Completed surveys were sealed in opaque envelopes and deposited into locked collection boxes placed in staff rest areas. The researcher retrieved all questionnaires in person at the end of the collection period. A mobile gift card (approximately USD 3.50) was provided after survey completion; contact information collected for this purpose was destroyed immediately after distribution and was not used in the analysis.

### 2.5. Data Analysis

Data were analyzed using IBM SPSS Statistics for Windows, version 27.0 (IBM Corp., Armonk, NY, USA). Descriptive statistics (frequencies, percentages, means, and standard deviations) were used to characterize the sample and study variables. Normality was confirmed by skewness (<|2|) and kurtosis (<|7|) values for all variables. Independent-samples *t*-tests and one-way ANOVA with Scheffé post hoc tests were used to examine differences in job esteem by demographic and job-related characteristics. Pearson’s correlation coefficients were calculated to examine bivariate relationships among the study variables.

Multiple linear regression analysis was conducted to identify factors associated with job esteem. Variables showing significant bivariate associations with job esteem were included as covariates, alongside the theory-driven predictors of organizational climate and role conflict. Pre-role training experience was dummy-coded (1 = yes, 0 = no [reference]). PA nursing experience was represented by two dummy variables: 3–<5 years (1 = yes, 0 = no) and ≥5 years (1 = yes, 0 = no), with <3 years as the reference category. Regression assumptions were evaluated: residual normality was assessed via a P-P plot and histogram of standardized residuals; homoscedasticity was confirmed by visual inspection of a scatterplot of standardized predicted values against residuals; independence of residuals was verified by the Durbin-Watson statistic; and multicollinearity was examined using tolerance and variance inflation factor (VIF) values.

### 2.6. Ethical Considerations

This study received approval from the Institutional Review Board of Keimyung University (IRB No. 40525-202501-HR-093-02). All participants provided written informed consent. Anonymity and confidentiality were maintained throughout data collection. Collected data were de-identified with unique codes, encrypted, and stored on a password-protected computer accessible only to the researcher. All data will be retained for three years and subsequently destroyed.

## 3. Results

### 3.1. Demographic and Job-Related Characteristics

Participants (*N* = 145) were predominantly female (62.8%, *n* = 91), with a mean age of 31.12 ± 4.30 years. Most held a bachelor’s degree (83.4%). Regarding organizational affiliation, 57.2% belonged to the nursing department and 42.8% to the medical department. The most common clinical area was surgical departments (46.9%), followed by internal medicine (29.0%) and other departments including emergency medicine, pediatrics, urology, and extracorporeal circulation (24.1%).

The mean total clinical experience was 6.65 ± 4.05 years, with 78.6% having ≥5 years. The mean physician assistant (PA) nursing experience was 4.26 ± 3.35 years; 40.0% had ≥5 years, 29.0% had 3–<5 years, and 31.0% had <3 years. Most participants held permanent positions (93.8%) and worked day shifts (55.2%). The primary motivation for becoming a PA nurse was formal appointment or transfer (77.9%). Most could take leave freely (80.0%), while only 18.6% (*n* = 27) had received any pre-role training prior to assuming PA duties (Table 1).

### 3.2. Levels of Organizational Climate, Role Conflict, and Job Esteem

The overall mean score for organizational climate was 2.59 ± 0.45 (range 1–4). Among the subscales, “relationships with medical staff” scored highest (2.93 ± 0.55), followed by “independent practice and support” (2.68 ± 0.46), “professional visibility” (2.68 ± 0.53), and “relationships with administrators” (2.19 ± 0.62).

The mean role conflict score was 3.38 ± 0.60 (range 1–5). “Work overload” was highest (3.59 ± 0.94), followed by “role ambiguity” (3.51 ± 0.69), “perceived incompetence” (3.19 ± 0.69), and “lack of cooperation” (3.12 ± 0.77).

The mean job esteem score was 3.71 ± 0.49 (range 1–5). Intrinsic professional dimensions scored relatively highly—“care role and expertise” (4.08 ± 0.69), “social trust and respect” (3.97 ± 0.73), and “professional self-awareness” (3.90 ± 0.55)—while extrinsic organizational dimensions were notably lower, with “organizational respect and recognition” at 3.10 ± 0.96 and “professional authority and future value” at 3.32 ± 0.86. All variables satisfied normality assumptions (skewness < |2|, kurtosis < |7|) (Table 2).

### 3.3. Differences in Job Esteem by Demographic and Job-Related Characteristics

Among all demographic and job-related characteristics examined, only PA nursing experience and pre-role training experience showed statistically significant differences in job esteem (Table 3).

PA nurses with less than 3 years of PA experience reported significantly higher job esteem (3.91 ± 0.46) than both the 3–<5 year group (3.61 ± 0.50) and the ≥5 year group (3.69 ± 0.51) (F = 4.94, *p* = 0.008; Scheffé post hoc). The mean difference between the <3 year and 3–<5 year groups was 0.30 points (Cohen’s d = 0.63, medium effect). PA nurses with pre-role training experience (3.96 ± 0.57) scored 0.31 points higher than those without (3.65 ± 0.46; t = 3.05, *p* = 0.003; Cohen’s d = 0.60, medium effect). No other characteristics showed significant differences.

### 3.4. Correlations Among Organizational Climate, Role Conflict, and Job Esteem

Organizational climate showed a significant positive correlation with job esteem (r = 0.48, *p* < 0.001), and role conflict showed a significant negative correlation with job esteem (r = −0.24, *p* < 0.001). Organizational climate and role conflict were also negatively correlated (r = −0.43, *p* < 0.001) (Table 4).

### 3.5. Factors Associated with Job Esteem

Multiple linear regression analysis was conducted with job esteem as the dependent variable (Table 5). Pre-role training experience (dummy-coded: 1 = yes, 0 = no [reference]) and PA nursing experience (two dummy variables: 3–<5 years and ≥5 years, both vs. <3 years as reference) were entered as covariates alongside the theory-driven predictors (organizational climate and role conflict).

The overall model was statistically significant (F = 9.64, *p* < 0.001), with an adjusted R^2^ of 0.23. Regression diagnostics confirmed the validity of the model: the Durbin-Watson statistic was 1.73 (indicating independent residuals), tolerance values ranged from 0.65 to 0.94 (all > 0.10), VIF values ranged from 1.06 to 1.54 (all < 10), and visual inspection of residual plots confirmed approximate normality and homoscedasticity of residuals.

Organizational climate showed the strongest association with job esteem (B = 0.44, 95% CI [0.26, 0.62], β = 0.40, *p* < 0.001), followed by pre-role training experience (B = 0.27, 95% CI [0.09, 0.45], β = 0.22, *p* = 0.003). Role conflict (B = −0.01, 95% CI [−0.14, 0.11], β = −0.02, *p* = 0.795), PA experience 3–<5 years vs. <3 years (B = −0.15, 95% CI [−0.34, 0.04], β = −0.14, *p* = 0.117), and PA experience ≥ 5 years vs. <3 years (B = −0.08, 95% CI [−0.27, 0.11], β = −0.08, *p* = 0.397) were not statistically significant.

## 4. Discussion

This study examined the associations of organizational climate and role conflict with job esteem among physician assistant (PA) nurses in South Korea. The findings indicate that organizational climate and pre-role training experience showed the strongest associations with job esteem in this sample, while role conflict did not demonstrate an independent association when organizational climate was accounted for. These findings carry implications for hospital management, workforce policy, and the broader international discussion on how to support nurses undertaking expanded clinical roles.

### 4.1. A Pattern Suggestive of Organizational Under-Recognition

The subscale analysis of job esteem revealed that PA nurses scored highly on intrinsic professional dimensions such as care role and expertise (4.08/5) and social trust and respect (3.97/5), but considerably lower on organizational respect and recognition (3.10/5). This pattern may reflect a gap between expanding responsibilities and institutional acknowledgment. Paralleling this, the lowest organizational climate subscale was relationships with administrators (2.19/4), suggesting limited administrative support and communication.

These findings are consistent with international literature documenting that nurses in expanded roles often experience a disconnect between clinical autonomy and organizational visibility [3,28]. However, it should be noted that this interpretation is based on subscale mean patterns rather than a direct measure of organizational recognition. Nonetheless, the convergent pattern across both instruments suggests that enhancing institutional acknowledgment—through representation in decision-making, transparent performance feedback, and equitable communication structures—may be a worthwhile avenue for supporting job esteem among PA nurses.

### 4.2. Organizational Climate as the Factor Most Strongly Associated with Job Esteem

Organizational climate showed the strongest association with job esteem (β = 0.40), consistent with prior findings among Korean PA nurses [10,16,23] and international evidence linking positive organizational climates to professional pride in nursing [17,18,29]. A one-unit increase in organizational climate score was associated with a 0.44-point higher job esteem score (95% CI: 0.26–0.62), representing a practically meaningful association given the 5-point scale of the outcome measure.

Comparisons with international settings, though illustrative rather than directly comparable given differences in healthcare systems and measurement contexts, provide useful reference points. Poghosyan et al. [15] reported that US nurse practitioners scored relatively higher on independent practice and support, likely reflecting well-established legal and institutional frameworks that support autonomous practice. By contrast, the moderate overall organizational climate score (2.59/4) and notably low administrator-relationship scores observed in this study may reflect the dual organizational structure in which Korean PA nurses are administratively affiliated with the nursing department but functionally supervised by the medical department [16,24]. This structural ambiguity may dilute both administrative advocacy and organizational belonging—conditions that may be amenable to institutional intervention.

### 4.3. Pre-Role Training: A Neglected but Potentially Relevant Factor

Pre-role training experience was significantly associated with higher job esteem (β = 0.22), with trained PA nurses scoring 0.31 points higher (Cohen’s d = 0.60, medium effect). This finding is noteworthy given that only 18.6% of participants had received any pre-role training—indicating that the vast majority transitioned into expanded roles without structured preparation.

This finding is consistent with Lee and Kim [24], who reported that trained PA nurses showed higher job satisfaction and lower role conflict. Internationally, the principle of preparing nurses for expanded roles before they assume them is well established: the United States requires formal graduate education and national certification for advanced practice nurses [30], Canada mandates master’s-level education with regulated scope of practice [31], and Australia emphasizes competency-based frameworks and educational accessibility [32]. While direct comparisons between these contexts and the Korean PA nurse role are limited by fundamental differences in credentialing systems, the shared finding that structured preparation is associated with better professional outcomes suggests that formalized pre-role education may help improve readiness and professional self-regard among Korean PA nurses.

### 4.4. Role Conflict and Its Non-Significant Independent Association

Role conflict was negatively correlated with job esteem at the bivariate level (r = −0.24, *p* < 0.001) but did not show a statistically significant independent association in the regression model (β = −0.02, *p* = 0.795). This finding warrants careful interpretation, as several explanations are plausible.

The most parsimonious explanation involves shared variance: organizational climate and role conflict were moderately correlated (r = −0.43), indicating that the aspects of role conflict most relevant to job esteem may overlap substantially with what the organizational climate construct already captures. Although the statistical indicators of multicollinearity were within acceptable limits (VIF < 1.54), conceptual overlap between unsupportive organizational conditions and perceived role conflict should be acknowledged. Future studies may benefit from modeling these constructs as partially overlapping dimensions of role-related organizational strain, or from examining mediation pathways in which organizational climate conditions shape role conflict perceptions, which in turn affect job esteem.

The findings are also compatible with the possibility that in settings where PA nurses perceive a supportive organizational climate, the association between role ambiguity and professional self-regard may be attenuated. This interpretation is consistent with Naseer et al. [33], who found that perceived organizational support moderated the relationship between role conflict and job outcomes. However, this study did not formally test moderation or mediation pathways, and the non-significance of role conflict may also reflect insufficient statistical power for detecting smaller independent effects or the possibility that role conflict relates to job esteem through indirect pathways (e.g., via burnout or job satisfaction) rather than directly. Future research using structural equation modeling would be needed to disentangle these mechanisms.

### 4.5. The Paradox of Declining Job Esteem with Experience

PA nurses with less than 3 years of PA experience reported significantly higher job esteem than more experienced colleagues (Cohen’s d = 0.63). This finding contrasts with research suggesting that career maturation typically strengthens professional identity [34]. Several explanations are plausible: early-career PA nurses may bring fresh enthusiasm and role expectations that have not yet been eroded by cumulative exposure to structural constraints—role ambiguity, inadequate authority, and insufficient institutional recognition—that become more salient over time [7,8]. This pattern may parallel the disillusionment trajectory documented in nursing workforce research [34] and underscores the need for sustained organizational investment in PA nurses beyond the onboarding period.

Although PA experience did not remain a significant predictor in the regression model when organizational climate was accounted for, the group-level differences observed in Table 3 suggest that career-stage dynamics merit further investigation, preferably through longitudinal designs that can capture trajectories of job esteem over time.

### 4.6. Explained Variance and Implications for Future Research

The regression model explained 23% of the variance in job esteem (adjusted R^2^). This indicates that organizational climate and training were meaningfully associated with job esteem; however, the majority of variance remains attributable to unmeasured factors. From a practical standpoint, a 23% explained variance in a cross-sectional study of a complex psychosocial outcome is within the range commonly observed in nursing workforce research [10,23]. Candidate variables for future investigation include individual psychological resources (self-efficacy, resilience, professional identity), organizational factors (leadership styles, interprofessional team climate, compensation adequacy), and system-level variables (legal status clarity, public perception of nursing roles). Mixed-methods designs incorporating qualitative exploration of PA nurses’ lived experiences would further enrich understanding of how job esteem is constructed and maintained in contexts of institutional ambiguity.

### 4.7. Limitations

Several limitations should be acknowledged. First, convenience sampling was used, and the sample was drawn exclusively from tertiary university hospitals in a single metropolitan area. Because the participating hospitals were all tertiary university-affiliated institutions in a single urban area, the findings may reflect organizational cultures specific to large teaching hospitals and may not generalize to non-tertiary settings, smaller hospitals, community clinics, or other regions. Self-selection bias inherent in voluntary participation may also have influenced the results. Second, the cross-sectional design precludes causal inference; the associations identified should be interpreted as correlational, and longitudinal studies would be needed to clarify temporal and directional relationships. Third, because all study variables were measured using self-report questionnaires collected at a single time point, common method variance cannot be ruled out. Fourth, PA nursing experience was treated as a categorical variable, which may obscure nonlinear relationships that could be detected with continuous or polynomial specifications. Fifth, pre-role training was assessed only as a dichotomous variable and did not capture differences in training duration, content, quality, or formal accreditation; future studies should characterize training experiences more precisely. Sixth, the study did not test potential mediating or moderating pathways between role conflict, organizational climate, and job esteem—relationships that could be explored through structural equation modeling in future research.

## 5. Conclusions

This study found that job esteem among physician assistant (PA) nurses was more strongly associated with organizational climate and pre-role training experience than with role conflict when these factors were considered simultaneously. In particular, the “relationships with administrators” subscale of the organizational climate showed the lowest score in this sample (2.19/4), and the “organizational respect and recognition” dimension of job esteem (3.10/5) was substantially lower than the intrinsic professional dimensions, suggesting that limited administrative engagement and organizational recognition—rather than overall workload alone—may represent a particularly modifiable target. Hospitals seeking to support PA nurses’ professional self-regard may benefit from enhancing administrator engagement and communication structures, and from establishing structured pre-role training programs. At the policy level, clarifying the legal scope of PA nursing practice and developing standardized educational pathways may help create conditions that support job esteem. These findings should also be considered against the backdrop of the broader physician shortage. In South Korea, persistent physician shortages and the recent disruption following the 2024 healthcare reform conflict have intensified the reliance on PA nurses to maintain hospital services [6,8], which can compromise care quality if expanded clinical responsibilities are not matched by adequate institutional support. Sustained role expansion under such conditions may erode the organizational climate by increasing workload, blurring task boundaries, and diluting administrative support, which in turn may threaten patient safety, continuity of care, and the long-term sustainability of the nursing workforce. Comparable concerns have been documented in other countries facing physician shortages and accelerated task-shifting [1,2,5], suggesting that organizational supports for nurses in physician-delegated roles are relevant not only to professional well-being but also to the overall quality of healthcare services. These findings may also be informative for other healthcare systems in which nursing role expansion is occurring faster than the development of supporting institutional structures.

## Figures and Tables

**Table 1:** Demographic and Job-Related Characteristics of Participants (*N* = 145).

Characteristic	Category	*n* (%)	M ± SD
Sex	Female	91 (62.8)	
	Male	54 (37.2)	
Age (years)	20–29	52 (35.9)	31.12 ± 4.30
	30–39	85 (58.6)	
	40–49	8 (5.5)	
Education	Associate degree	14 (9.7)	
	Bachelor’s degree	121 (83.4)	
	Master’s degree	10 (6.9)	
Departmental affiliation	Medical department	62 (42.8)	
	Nursing department	83 (57.2)	
Clinical department	Surgical	68 (46.9)	
	Internal medicine	42 (29.0)	
	Other *^a^*	35 (24.1)	
Total clinical experience (years)	<3	9 (6.2)	6.65 ± 4.05
	3–<5	22 (15.2)	
	≥5	114 (78.6)	
PA nursing experience (years)	<3	45 (31.0)	4.26 ± 3.35
	3–<5	42 (29.0)	
	≥5	58 (40.0)	
Employment type	Permanent	136 (93.8)	
	Contract	9 (6.2)	
Work pattern	Day shift	80 (55.2)	
	Rotating shifts	65 (44.8)	
Motivation for PA role	Appointed/transferred	113 (77.9)	
	Avoid rotating shifts	25 (17.2)	
	Professional development	7 (4.8)	
Ability to take leave freely	Yes	116 (80.0)	
	No	29 (20.0)	
Pre-role training experience	Yes	27 (18.6)	
	No	118 (81.4)	

*^a^* Emergency medicine, pediatrics, urology, extracorporeal circulation.

**Table 2:** Levels of Organizational Climate, Role Conflict, and Job Esteem (*N* = 145).

Variable	Scale Range	M ± SD	Skewness	Kurtosis
Organizational climate	1–4	2.59 ± 0.45	0.66	0.98
Professional visibility		2.68 ± 0.53	0.09	0.50
Relationships with administrators		2.19 ± 0.62	0.72	0.60
Relationships with medical staff		2.93 ± 0.55	−0.43	0.29
Independent practice and support		2.68 ± 0.46	0.33	0.91
Role conflict	1–5	3.38 ± 0.60	−0.57	0.43
Role ambiguity		3.51 ± 0.69	−0.71	0.78
Perceived incompetence		3.19 ± 0.69	−0.17	−0.37
Work overload		3.59 ± 0.94	−0.80	0.34
Lack of cooperation		3.12 ± 0.77	−0.54	0.34
Job esteem	1–5	3.71 ± 0.49	−0.33	0.83
Professional self-awareness		3.90 ± 0.55	0.01	−0.64
Professional competence		3.74 ± 0.58	0.03	0.02
Care role and expertise		4.08 ± 0.69	−0.60	−0.07
Social trust and respect		3.97 ± 0.73	−0.57	0.23
Organizational respect and recognition		3.10 ± 0.96	−0.29	−0.21
Professional authority and future value		3.32 ± 0.86	−0.28	−0.26

**Table 3:** Differences in Job Esteem by Characteristics (*N* = 145).

Characteristic	Category	M ± SD	t/F (*p*)	Post Hoc
Sex	Female	3.73 ± 0.48	−0.63 (0.529)	
	Male	3.67 ± 0.52		
Age (years)	20–29	3.77 ± 0.54	0.28 (0.750)	
	30–39	3.71 ± 0.49		
	40–49	3.64 ± 0.38		
Departmental affiliation	Medical dept.	3.78 ± 0.45	1.44 (0.149)	
	Nursing dept.	3.66 ± 0.52		
Total clinical experience (years)	<3	3.90 ± 0.35	2.74 (0.067)	
	3–<5	3.84 ± 0.53		
	≥5	3.68 ± 0.50		
PA experience (years)	<3 *^a^*	3.91 ± 0.46	4.94 (0.008)	*^a^* ^>^ ^*b*,^ *^a^* ^>^ *^c^* ^†^
	3–<5 *^b^*	3.61 ± 0.50		
	≥5 *^c^*	3.69 ± 0.51		
Pre-role training	Yes	3.96 ± 0.57	3.05 (0.003)	
	No	3.65 ± 0.46		

† Scheffé post hoc test. The <3 year group (*^a^*) scored significantly higher than the 3–<5 year group (*^b^*) and the ≥5 year group (*^c^*).

**Table 4:** Correlations among Study Variables (*N* = 145).

Variable	Organizational Climate	Role Conflict	Job Esteem
Organizational climate	1		
Role conflict	−0.43 (*p* < 0.001)	1	
Job esteem	0.48 (*p* < 0.001)	−0.24 (*p* < 0.001)	1

**Table 5:** Multiple Linear Regression Analysis: Factors Associated with Job Esteem (*N* = 145).

Variable	B	SE	β	t	*p*	95% CI for B
(Constant)	2.68	0.44		6.09	<0.001	[1.81, 3.55]
Organizational climate	0.44	0.09	0.40	4.89	<0.001	[0.26, 0.62]
Pre-role training (yes vs. no) *^a^*	0.27	0.09	0.22	2.99	0.003	[0.09, 0.45]
Role conflict	−0.01	0.06	−0.02	−0.26	0.795	[−0.14, 0.11]
PA experience 3–<5 yrs vs. <3 yrs *^b^*	−0.15	0.10	−0.14	−1.58	0.117	[−0.34, 0.04]
PA experience ≥5 yrs vs. <3 yrs *^b^*	−0.08	0.10	−0.08	−0.85	0.397	[−0.27, 0.11]

F = 9.64, *p* < 0.001, R^2^ = 0.26, adjusted R^2^ = 0.23, Durbin-Watson = 1.73. *^a^* Reference: no training experience. *^b^* Reference: <3 years of PA experience.

## Data Availability

The data presented in this study are available on request from the corresponding author due to privacy and ethical restrictions.
